# Coordinated Development and Spatiotemporal Evolution Trends of China’s Agricultural Trade and Production from the Perspective of Food Security

**DOI:** 10.3390/foods14142538

**Published:** 2025-07-20

**Authors:** Yueyuan Yang, Chunjie Qi, Yumeng Gu, Cheng Gui

**Affiliations:** 1 College of Economics and Management, Huazhong Agriculture University, Wuhan 430070, China; yangyueyuan@webmail.hzau.edu.cn (Y.Y.); guyumeng@webmail.hzau.edu.cn (Y.G.); guicheng@webmail.hzau.edu.cn (C.G.); 2 Hubei Rural Development Research Center, Huazhong Agriculture University, Wuhan 430070, China; 3 Institute of Finance and Economics, Wuhan City Polytechnic, Wuhan 430070, China

**Keywords:** agricultural product trade, agricultural product production, coupling coordination, spatial effects

## Abstract

Ensuring food security necessitates a high level of coordinated development between agricultural trade and production. Based on China’s provincial panel data from 2010 to 2023, this study constructs an evaluation index system for agricultural trade and production, employing an entropy-weighted TOPSIS model to measure their development levels. On this basis, a coupling coordination degree model and Moran’s I indices are used to analyze the coordinated development level’s temporal changes and spatial effects. The research finds that the development levels of China’s agricultural trade and production show an upward trend but currently still exhibit the pattern of higher levels in Eastern China and lower levels in Western China. The coupling coordination level between them demonstrates an increasing trend, yet the overall level remains relatively low, with an average value of only 0.445, consistently staying in a marginal disorder “running-in stage” and spatially presenting a distinct “east-high–west-low” stepped distribution pattern. Furthermore, from a spatial perspective, the Global Moran’s index decreased from 0.293 to 0.280. The coupling coordination degree of agricultural trade and production in China generally exhibits a positive spatial autocorrelation, but this effect has been weakening over time. Most provinces show spatial clustering characteristics of high–high and low–low agglomeration in local space, and this feature is relatively stable. Building on these insights, this study proposes a refinement of the coordination mechanisms between agricultural trade and production, alongside the implementation of differentiated regional coordinated development strategies, to promote the coupled and coordinated advancement of agricultural trade and production.

## 1. Introduction

The foundation for achieving agricultural sustainable development and ensuring food security lies in continuously enhancing the coupling coordination level between agricultural trade and production. Since reform and opening-up, China has actively utilized both domestic and international markets and resources to optimize the allocation of agricultural resources, appropriately increasing imports of domestically scarce agricultural products, thereby forging a distinctive path toward food security with Chinese characteristics. However, in recent years, the contradiction between the expansion of agricultural trade and domestic production has become increasingly prominent, with excessively rapid import growth for certain products significantly impacting domestic agriculture. According to data from China’s General Administration of Customs, in 2024, China’s cumulative grain imports reached 158 million tons, with corn imports exceeding tariff quotas for five consecutive years, and beef imports reached 2.87 million tons. Large-scale imports, coupled with declining domestic demand, have directly pressured domestic agricultural prices downward, severely undermining farmers’ enthusiasm for cultivation and breeding and even threatening China’s agricultural industry security. In response to these real challenges, the 2024 Central Economic Work Conference and the 2025 Central No. 1 Document clearly put forward the policy orientation of “improving the coordination mechanism between agricultural trade and production”, calling for the establishment of a coordinated framework between the two. This highlights the extreme urgency of addressing their coordinated development at the national strategic level and provides a direct policy basis and practical starting point for this research. Accordingly, scientifically examining the coupling relationship and spatiotemporal evolution patterns of agricultural trade and production development, exploring their spatial effects, and promoting benign interactions and coordinated development between them can provide crucial safeguards for food security while offering strong endogenous momentum for high-quality agricultural development.

Scholars have conducted extensive research on the relationship between agricultural trade and production development. Traditional comparative advantage theory emphasizes that agricultural production relies on factor endowments such as land, labor, and water, directly determining agricultural trade patterns; for instance, land-abundant countries export land-intensive products such as soybeans and corn while importing labor-intensive products such as vegetables, fruits, and aquatic products [1,2]. Research by Andrianarimanana and Pu (2021) found that improvements in agricultural technology innovation in Sub-Saharan Africa have increased world trade volume [3]. Ramesh’s study (2021) confirmed that agricultural production significantly promotes the growth of agricultural trade by determining the availability and specialization of agricultural products [4]. Ferguson & Gars (2020) further point out that agricultural production shocks, such as droughts, floods, or pest infestations, may lead to substantial fluctuations in the quantity and quality of agricultural output, with such shocks not only affecting domestic markets but also significantly influencing the trade volume and unit value of agricultural products [5]. However, unlike the direct promotional effect of agricultural production on trade, the impact of agricultural trade on production is far more complex. On the one hand, most scholars acknowledge the positive role of agricultural trade in production [6,7,8], arguing that it enhances agricultural technical efficiency and output through comparative advantage, increasing returns to scale, and technology transfer [9,10], and that spillover effects from both imports and exports increase the productivity of traditional crops [11]. Specifically, Bai et al. (2021) found that agricultural trade improves land and nitrogen use efficiency, effectively conserving farmland and fertilizer resources [12]. Farrokhi & Pellegrina (2023) further demonstrated that trade in agricultural inputs is equally critical, with cost reductions and sectoral productivity growth significantly driving agricultural productivity gains [13]. On the other hand, studies also reveal that agricultural trade may negatively affect production. Trade liberalization accelerates the transfer of agricultural labor, leading to the loss of agricultural workers with higher human capital, thereby inhibiting productivity growth [14]. Simultaneously, agricultural trade drives agricultural expansion, exacerbating agricultural water scarcity [15], increasing greenhouse gas emissions [16], threatening biodiversity while intensifying habitat loss [17], and ultimately undermining food autonomy at local and national levels [18,19]. However, previous literature on agricultural trade and production has primarily focused on the unidirectional impact mechanisms, either of agricultural trade on production or vice versa. Systematic empirical research on the interactive relationship, coupling coordination status, and spatiotemporal evolution patterns between the two remains insufficient. In reality, agricultural trade and production constitute a complex system characterized by close interconnections and dynamic evolution, whose coordinated development therefore requires in-depth exploration.

Addressing this research gap, the core objective of this paper is to systematically measure the coupling coordination relationship between agricultural trade and production in China using quantitative methods and reveal its spatiotemporal evolution patterns and spatial effects. The main contributions of this paper are as follows: First, this is the first study to provide systematic empirical evidence on the interactive relationship between agricultural trade and production from the perspective of coupling coordination, filling the gap left by existing literature that mostly focuses on unidirectional impacts. Second, it reveals the spatiotemporal characteristics and spatial associations of their coordinated development, providing a scientific basis for understanding regional differences and formulating differentiated policies. One of the few precedents is the study of Zhu Jing (2025), who proposed policy recommendations to improve the coordination mechanism of agricultural trade and production from the perspectives of agricultural imports, production, and exports, based on China’s current domestic and international economic environment [20]. Unlike that research, we not only provide policy recommendations but more importantly, for the first time, empirically measure and reveal the coordinated linkage effects and spatial patterns between agricultural trade and production using coupling coordination models. This provides a direct quantitative assessment tool and decision-making support basis for the policy goal of “improving the coordination mechanism”. To our knowledge, this is the first empirical paper to systematically study the coordinated relationship between agricultural trade and production.

This study holds significant theoretical and practical implications. Theoretically, it enriches the application of agricultural economic systems theory and complex systems theory in the study of the interactive relationship between agricultural trade and production. Practically, the results of this study can provide a precise quantitative basis and spatial decision-making references for central and local governments to formulate and improve the “coordination mechanism between agricultural trade and production”, thereby contributing to the realization of national food security strategies and high-quality agricultural development goals. For example, regions with low coordination levels can be prioritized for policy support, while spatial spillover effects suggest the need for strengthened regional collaborative governance. The conclusions of this study also have reference value for agricultural producers and operators to understand market environments, adjust production structures, and mitigate risks.

This paper also contributes to enriching and complementing food security research. For instance, Wang et al. (2023) explored the impact of agricultural trade relationship stability, arguing that stable global agricultural trade relations are essential for regional and global food production stability and food security [21]. Aremu et al. (2021) emphasized that agricultural production is critical to food security, noting that strategies such as enhancing youth agricultural entrepreneurships, improving land access, and promoting innovative research can boost agricultural productivity and achieve inclusive growth [22]. Unlike our paper, these studies focus on food security assurance paths from the single dimension of promoting agricultural trade or enhancing agricultural production. Here, we innovatively consider the spatial characteristics and coupling coordination relationship between agricultural trade and production, demonstrating that improving the coordination level and optimizing the spatial patterns of the two can become an important driving force and effective pathway for ensuring national food security and enhancing agricultural industry resilience.

## 2. Research Theoretical Analysis Framework

### 2.1. Mechanism Analysis of Coupling Coordination Between Agricultural Trade and Production

Ensuring food security requires coordinated, orderly, and mutual progress in agricultural trade and production development. It must be noted, however, that the relationship between agricultural trade and production embodies a dialectical unity of opposites—a protracted, dynamic historical evolution. Specifically, on the one hand, agricultural trade provides momentum and safeguards for production development, though this outcome is by no means inevitable. Its actual impact is highly contingent upon the specific nature of trade, the operational mechanisms, the policy environment, and the intrinsic conditions of production systems, rendering trade’s influence on production inherently uncertain. Trade can enhance domestic production efficiency by introducing advanced agricultural technologies and management practices [23,24], while trade demand compels domestic production restructuring toward competitive sectors, driving quality-efficiency improvements, supply chain integration, and value-added enhancement. Simultaneously, agricultural trade may also transmit international risks, squeeze domestic markets, and disrupt local production. In essence, trade can drive production growth through market expansion, resource optimization, and technology spillovers, while conversely undermining production sustainability via price shocks, structural imbalances, and market volatility. On the other hand, agricultural production objectively forms the foundation of trade development—a universally acknowledged fact. Domestic production scale establishes the material foundation for trade volume; production efficiency and quality determine trade competitiveness; production structure optimization propels trade transformation; and production sustainability influences long-term trade stability. Ultimately, production directly shapes trade structure and competitiveness through factor allocation, capacity scale, efficiency upgrading, and policy synergy. In summary, as dual pillars of food security, agricultural trade and production do not exist in simple parallel but rather exhibit an intrinsically unified, coupling-coordinated dialectical relationship. Their coupling coordination level hinges profoundly upon mutual transformation, interdependence, and reciprocal spillovers. When trade and production develop coordinately, positive interactions between subsystems progressively enhance the effective supply capacity for grain and vital agricultural products, thereby safeguarding food security. Conversely, uncoordinated development exacerbates supply–demand contradictions, increasingly jeopardizing food security. Thus, coupling-coordinated development achieves guaranteed food security through the bidirectional interaction of “using trade to promote production and using production to stabilize trade”.

### 2.2. Construction of an Evaluation Index System

#### 2.2.1. Agricultural Product Trade

Current research on constructing agricultural trade index systems remains limited. Tyszynski (1951) proposed decomposing export fluctuations in the Constant Market Share (CMS) model into three components: the structural effect, competitiveness effect, and cross effect [25]. The structural effect refers to changes in an exporter’s export value caused by shifts in the import volume or structure of specific products in importing countries; the competitiveness effect denotes changes in export value resulting from variations in a country’s export competitiveness for specific products; and the cross effect captures the combined influence of the competitiveness and structural effects. Subsequent studies by Leamer et al. (1970), Jepma (1986), and Milana (1988) refined this framework [26,27,28].

Building upon Tyszynski (1951) [25] and related research, this study constructs an agricultural trade evaluation index system (Table 1) with 13 indicators across three dimensions: trade scale, trade structure, and trade competitiveness. Trade scale, comprising total agricultural trade value, agricultural export value, agricultural import value, and agricultural trade balance, forms the foundation of trade development. Trade structure, serving as the driving force, incorporates indicators from both product and market dimensions. Product structure includes export-oriented indicators (processed agricultural product export share and agricultural sideline product export share) and import-oriented indicators (primary product import share and animal product import share). Market structure is evaluated through export and import market diversification indices. Trade competitiveness, representing the core dimension, includes export and import competitiveness [29], measured by the trade competitiveness index and export and import market shares.

#### 2.2.2. Agricultural Production

Numerous researchers have assessed agricultural production using diverse indicators. Some scholars employ single-dimensional metrics such as average agricultural yield or yield per unit area [30,31,32]. Others establish multidimensional evaluation systems; for instance, Stöckle et al. (1994) evaluated the relative sustainability of agricultural production systems through nine attributes—profitability, productivity, soil quality, water quality, air quality, energy efficiency, fish/wildlife habitat, quality of life, and social acceptance [33]. Walters et al. (2016) categorized production systems into three sectors: crop cultivation, livestock farming, and integrated crop–livestock systems [34]. Omokaro (2025) further incorporated crop production, livestock production, land resources, and crop yields into the agricultural production framework [35].

Drawing on these studies, this paper extracts key indicators across three core dimensions: production scale, production structure, and production efficiency. Production scale reflects the aggregate supply and commercialization capacity, encompassing both input and output dimensions. Input scale indicators include agricultural land area, water usage area, labor force size, total machinery power, and technology investment, objectively measuring agricultural production potential and foundational output capacity. Output scale indicators comprise total agricultural, forestry, animal husbandry, and fishery output value; value-added in agriculture, forestry, animal husbandry, and fisheries; and grain output, reflecting actual agricultural production achievements. Production structure mirrors supply composition and quality, gauged by indicators including cash crop sown area ratio and forestry output value share. Production efficiency evaluates input–output effectiveness and competitiveness through land and labor productivity and resource utilization efficiency, measuring system efficacy in utilizing land, labor, and capital resources—forming the basis for domestic and international trade competitiveness. The detailed indicator system is presented in Table 1.

## 3. Materials and Methods

### 3.1. Materials

This study utilizes panel data from 31 provincial-level regions in China (excluding Hong Kong, Macao, and Taiwan) from 2010 to 2023 as the research sample. Agricultural trade data were obtained from the General Administration of Customs database; agricultural production data were sourced from the National Database of the National Bureau of Statistics (NBS) website, China Statistical Yearbook, and China Rural Statistical Yearbook. Individual missing data were supplemented by searching the official websites of the Ministry of Agriculture and Rural Affairs and provincial agricultural departments.

### 3.2. Methods

**Figure 1:**
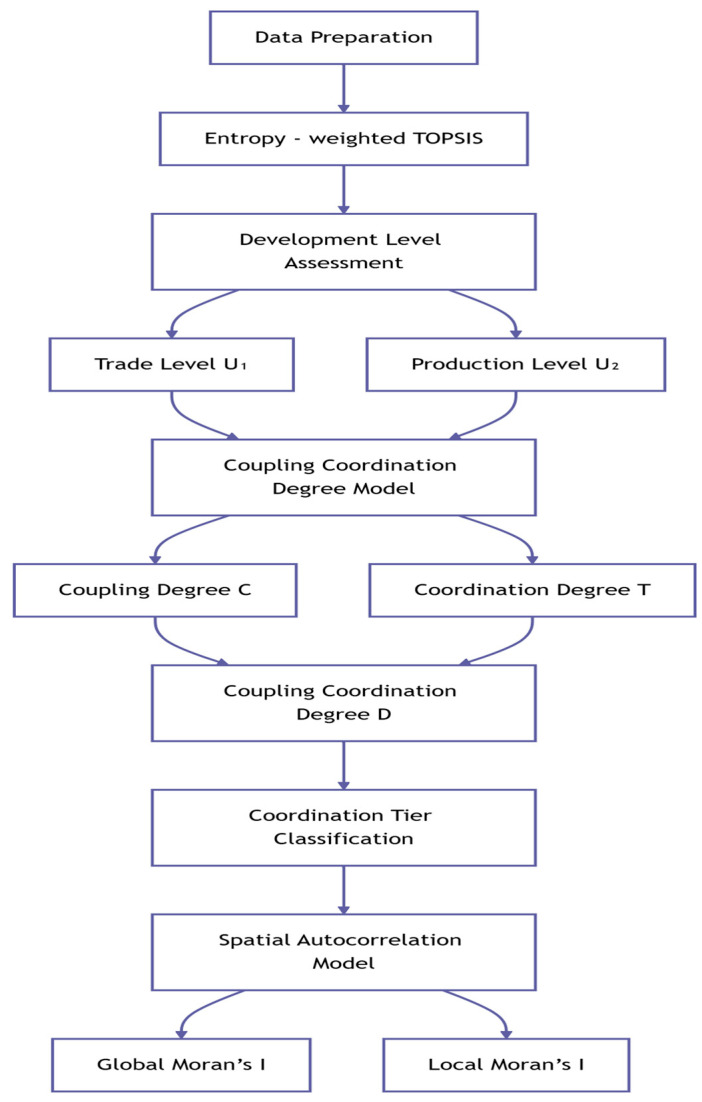
Flowchart of research methodology.

This study employs a multistage econometric analysis framework to systematically assess the coordinated relationship between agricultural trade and production. The research methodology is illustrated in Figure 1. Initially, based on the raw data of agricultural trade and production, the basic framework of the entropy-weighted TOPSIS model is constructed. Through key steps such as data standardization, weight assignment using the entropy-weighted method, and the construction of a weighted matrix, the development levels of trade and production are calculated. Subsequently, the calculated development levels are input into the coupling coordination degree model to successively measure the coupling degree (C), coordination degree (T), and coupling coordination degree (D). The robustness of the model is verified through parameter sensitivity analysis. Finally, based on the coupling coordination degree (D), a spatial autocorrelation analysis is conducted using a spatial autocorrelation model, and the Global Moran’s index and Local Moran’s index are employed to identify spatial agglomeration characteristics, thereby systematically completing the multidimensional measurement of the coordinated relationship between agricultural trade and production.

#### 3.2.1. The Entropy-Weighted TOPSIS Model

The entropy-weighted TOPSIS model integrates the entropy-weighted method and the TOPSIS technique, operating on the principle of first determining indicator weights via entropy weighting before employing TOPSIS to calculate the proximity to optimal performance levels. Following Habib et al. (2024) [36], this study applies the entropy-weighted TOPSIS model to evaluate the development levels of agricultural trade and production. The procedural steps are as follows:

Step 1: Data standardization. Construct an initial evaluation matrix for the agricultural trade and production indicator system with m provinces and n evaluation indicators:

(1) $$X=\left[\begin{array}{cccc} x_{11} & x_{12} & \ldots & x_{1n}\\ x_{21} & x_{22} & \ldots & x_{2n}\\ \vdots & \vdots & \vdots & \vdots \\ x_{m1} & x_{m2} & \ldots & x_{mn} \end{array}\right]$$

Dimensionless processing of the initial matrix is performed using the following transformation formulas for positive and negative indicators:

For positive indicators:

(2) $$Z_{ij}=\frac{x_{ij}-\min x_{ij}}{\max x_{ij}-\min x_{ij}}$$

For negative indicators:

(3) $$Z_{ij}=\frac{\max x_{ij}-x_{ij}}{\max x_{ij}-\min x_{ij}}$$

We obtain the standardized decision matrix Z.

(4) $$Z={\left[Z_{ij}\right]}_{m\times n}$$

Step 2: Calculate indicator weights. Determine the proportion of each indicator:

(5) $$r_{ij}=\frac{z_{ij}}{\sum_{i=1}^{m}z_{ij}}$$

Calculate the information entropy for each indicator:

(6) $$e_{j}=-\frac{\sum_{i=1}^{m}\left(r_{ij}\times \ln r_{ij}\right)}{\ln m}$$

Calculate the weights for each indicator:

(7) $$W_{j}=\frac{\left(1-e_{i}\right)}{\sum_{j=1}^{n}\left(1-e_{i}\right)}$$

Step 3: Construct the standardized weighted decision matrix. Multiply the standardized decision matrix by corresponding indicator weights to generate the weighted matrix:

(8) $$V=W\times Z={\left[v_{ij}\right]}_{mn}$$

Step 4: Determine positive ($V^{+}$) and negative ($V^{-}$) ideal solutions:

(9) $$V^{+}=\left\{\max v_{ij}|i=1,2,\cdots ,n\right\}=\left[v_{1}^{+},v_{2}^{+},\cdots ,v_{n}^{+}\right]$$

(10) $$V^{-}=\left\{\max v_{ij}|i=1,2,\cdots ,n\right\}=\left[v_{1}^{-},v_{2}^{-},\cdots ,v_{n}^{-}\right]$$

Step 5: Calculate the Euclidean distances from each evaluation object to the positive and negative ideal solutions:

(11) $$D_{j}^{+}=\sqrt{\sum_{i=1}^{m}{\left(v_{ij}-v_{i}^{+}\right)}^{2}}$$

(12) $$D_{j}^{-}=\sqrt{\sum_{i=1}^{m}{\left(v_{ij}-v_{i}^{-}\right)}^{2}}$$

Step 6: Calculate the relative closeness degree $U_{j}$; the value of $U_{j}$ ranges within [0, 1], where a higher value indicates a greater development level of agricultural trade and production in the province:

(13) $$U_{j}=\frac{D_{j}^{-}}{D_{j}^{+}+D_{j}^{-}}$$

#### 3.2.2. Coupling Coordination Degree Model

The coupling coordination degree model is primarily employed to assess coordinated development among multiple systems. Drawing on existing research [37,38], this study adopts this model to analyze the coupling coordination level between agricultural trade and production. The specific procedure is as follows:

First, calculate the coupling degree between agricultural trade and production:

(14) $$C=2{\left[\left(U_{1}U_{2}\right)/{\left(U_{1}+U_{2}\right)}^{2}\right]}^{\frac{1}{2}}$$

Then, calculate the coordination degree between agricultural trade and production:

(15) $$T=\alpha U_{1}+\beta U_{2}$$

where α and β are undetermined weight coefficients satisfying α+β=1. Considering the equally important status of agricultural trade and production in this study, we set α=β=0.5. The hypothesis is based on the following rational grounds. First, system symmetry: In the agricultural system, trade and production are interdependent and mutually driving core components. Production is the foundation of trade, while trade is the channel for realizing the value of production. The two hold theoretically equal status in the industrial chain. Second, avoiding subjective bias: In the absence of prior theoretical or empirical evidence supporting the dominance of one system over the other, equal weighting is a common practice to reduce subjective intervention and maintain analytical neutrality.

To verify the robustness of the equal-weighting assumption, this paper further conducts a sensitivity analysis. By adjusting the weight combinations (α=0.4, β=0.6; α=0.3, β=0.7; α=0.6, β=0.4; α=0.7, β=0.3) and recalculating the coupling coordination degree, we compare the results with the original findings. As shown in Table 2, the impact of weight changes on the coupling coordination degree is minimal, with the fluctuation of the national coupling coordination degree of agricultural trade and production being less than 5%. The main conclusions remain unchanged. This indicates that the equal-weighting assumption is reasonable in this study.

Finally, calculate the coupling coordination degree for agricultural trade and production:

(16) $$D=\sqrt{C\times T}$$

The value range of D is [0, 1]. A higher D indicates a stronger benign coordination between the two systems. The coupling coordination between agricultural trade and production is classified into ten grades and three major development stages (see Table 3): when 0≤D≤0.4, it is the antagonistic stage, indicating low coordination between trade and production within the region; when 0.4<D≤0.7, it is the running-in stage, reflecting a transition toward coordination though overall coupling effects remain weak; when 0.7<D≤1, it is the coordinated stage, signifying high synergy with achieved coupling coordination.

#### 3.2.3. Spatial Autocorrelation Model

The spatial autocorrelation model is employed to analyze the spatial characteristics of the coupling coordination degree between agricultural trade and production systems, encompassing both global and local spatial autocorrelation methods. For global spatial autocorrelation, the Global Moran’s I index measures the overall spatial dependence of the two systems, calculated as follows:

(17) $$I=\frac{\sum_{i=1}^{n}\sum_{j=1}^{n}w_{ij}\left(D_{i}-\bar{D}\right)\left(D_{j}-\bar{D}\right)}{\frac{1}{n}\sum_{i=1}^{n}{\left(D_{i}-\bar{D}\right)}^{2}\sum_{i=1}^{n}\sum_{j=1}^{n}w_{ij}}$$

In Formula (17), I denotes the Global Moran’s I index; $D_{i}$ and $D_{j}$ represent the coupling coordination degrees of the two systems in provinces i and j, respectively; $\bar{D}$ is the mean value; and $w_{ij}$ denotes the economic-distance spatial weight matrix. I ranges from [−1, 1], where I>0 indicates a positive spatial autocorrelation in the coupling coordination degree of agricultural trade and production, I<0 signifies a negative spatial autocorrelation, and I=0 suggests no significant spatial correlation.

For local spatial autocorrelation, the local indicators of spatial association (LISA) measure spatial dependence between a given province and its neighbors, calculated as follows:

(18) $$I_{i}=\frac{\left(D_{i}-\bar{D}\right)}{\frac{1}{n}\sum_{i=1}^{n}{\left(D_{i}-\bar{D}\right)}^{2}}\sum_{j=1}^{n}w_{ij}\left(D_{j}-\bar{D}\right)$$

In Formula (18), $I_{i}$ represents the Local Moran’s I index; the meanings of all other variables align with the technical specifications of the Global Moran’s I. $I_{i}>0$ indicates that the coupling coordination degree of a province is spatially homogeneous with its neighbors, demonstrating positive spatial autocorrelation, while $I_{i}<0$ signifies spatial heterogeneity between the province and its surrounding provinces, indicating negative spatial autocorrelation.

## 4. Results and Discussion

### 4.1. Spatiotemporal Analysis of China’s Agricultural Trade and Production Development Levels

**Figure 2:**
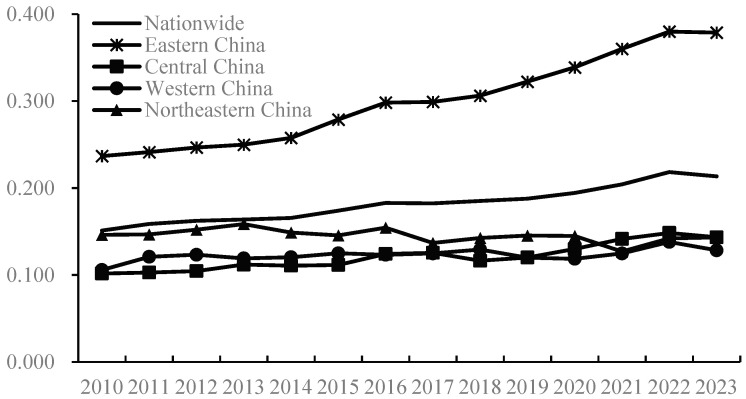
Development level of agricultural trade from 2010 to 2023.

**Figure 3:**
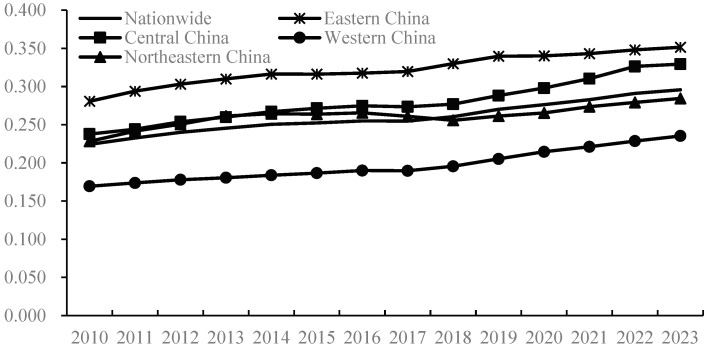
Development level of agricultural production from 2010 to 2023.

This study employs the entropy-weighted TOPSIS model to measure China’s agricultural trade and production development levels from 2010 to 2023 (Figure 2 and Figure 3). Overall, both China’s agricultural trade and production development levels exhibited a general upward trend during this period, rising from 0.151 and 0.224 to 0.213 and 0.296, respectively, indicating continuous improvement in both areas, which aligns with the actual progress of China’s agricultural economy. Comparatively, the development level of agricultural production has been higher than that of agricultural trade, with the former averaging 0.259 and the latter only 0.182. Nevertheless, it is undeniable that both remain relatively low with significant room for future growth. Further analysis of the growth trends reveals that the annual average growth rate of China’s agricultural trade development level was 2.69%, surpassing the 2.15% growth rate of agricultural production development during the same period, meaning that the development speed of agricultural trade nationwide outpaced that of agricultural production. This phenomenon can be attributed to China’s aggressive promotion of high-level agricultural opening-up strategies in recent years, which have accelerated the integration of domestic agriculture with global agricultural development, significantly expanded the degree of openness in the agricultural sector, and continuously optimized trade structures, thereby strongly driving the rapid development of agricultural trade. Currently, China has emerged as the world’s largest agricultural importer, fifth-largest exporter, and consistently maintained its position as the second-largest agricultural trading nation.

From a regional perspective, China’s agricultural trade and production development levels exhibit significant spatial imbalances, with Eastern China overall outperforming Northeast, Central, and Western China. In terms of agricultural trade, development levels exhibit the following pattern, showing a monotonic decline from east to west: Eastern China > Northeastern China > Central and Western China. Notably, the development gap between Eastern and Western China widened substantially from 0.131 in 2010 to 0.250 in 2023, indicating a continuous broadening of regional disparities and increasingly prominent imbalances in trade development. This divergence originates from Eastern China’s first-mover advantages in export-oriented agriculture, while Central and Western China face structural constraints in infrastructure, industrial chain integration, and institutional innovation. Regarding agricultural production, the development level also exhibits an “east-high–west-low” pattern. From 2010 to 2023, the average agricultural production indices for Eastern, Central, Northeastern, and Western China stood at 0.322, 0.279, 0.261, and 0.197, respectively. Eastern China significantly exceeds the national average; central and northeastern regions approximate it; and Western China lags markedly. This reflects a severe spatial inequity from east to west, attributable to Eastern China’s superior infrastructure, farmer education, economic development, and agricultural R&D capabilities. Meanwhile, Western China experiences sluggish growth in agricultural production development, trailing the national average and ranking last among the four regions. This is closely related to Western China‘s weak economic foundation, backward agricultural technologies, inadequate digitalization, and underdeveloped factor markets.

### 4.2. Spatiotemporal Analysis of the Coupling Coordination Between China’s Agricultural Trade and Production

**Figure 4:**
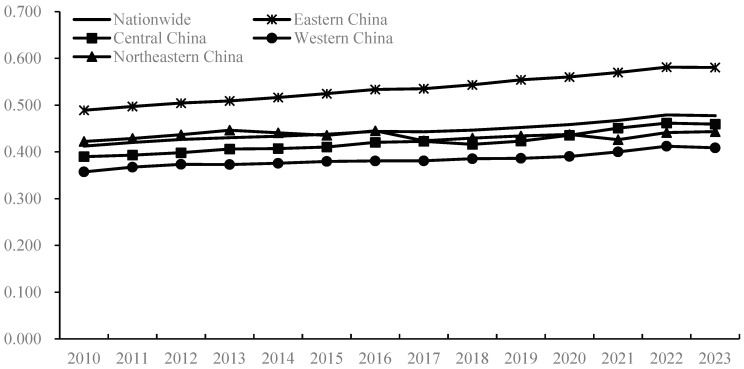
Coupling coordination level of agricultural trade and production at the national level and in the four major regions.

The coupling coordination degree between China’s agricultural trade and production was calculated using the coupling coordination degree model, with the results shown in Figure 4. Nationally, from 2010 to 2023, the coupling coordination degree between China’s agricultural trade and production demonstrated a gradual upward trajectory, increasing from 0.413 to 0.477, with an average annual growth rate of 1.13%. This indicates that China has achieved relatively favorable outcomes in the mutual promotion and coordinated development of agricultural trade and production, with the overall effect in safeguarding the effective supply of grain and important agricultural products being positive. It should be noted, however, that the current level of coupling coordination is relatively low, with an average value of only 0.445, remaining in the quasi-disorder “running-in stage”, signifying considerable distance from full coordination. Regionally, during the study period, the coupling coordination degree in all four major regions showed steady growth.

Among them, Eastern China is in an absolutely leading position, with its coupling coordination degree being significantly higher than that of other regions, rising from 0.489 in 2010 to 0.581 in 2023, undergoing an evolution from “on the verge of imbalance” to “barely coordinated”, being the only region in the country to have reached this latter stage. The coupling coordination degree level in the northeast region ranks second, with an average value of 0.435, but its growth rate is slow, with an average annual rate of only 0.37%, and it has long been at the level of being on the verge of imbalance. This indicates that there are still significant obstacles in the process of promoting the coupling coordination between agricultural trade and production in the northeast region, which may be related to slow economic development and the agricultural industrial structure being overly dependent on resource endowments, leading to a single structure and path dependence. The coupling coordination degree levels in the central and western regions rank third and fourth, respectively, with the average coupling coordination degree rising from 0.390 and 0.357 in 2010 to 0.459 and 0.409 in 2023, respectively, showing an overall shift from mild imbalance to being on the verge of imbalance, and from the antagonistic stage to the running-in stage. In summary, the coupling and coordination trend between China’s agricultural trade and production has become relatively evident at this stage, but the characteristic of “imbalance” in the coupling coordination among regions is obvious.

Provincially, the coupling coordination degree between agricultural trade and production strengthened across all provinces during 2010–2023, yet significant inter-provincial disparities persisted (Table 4). Temporally, the coupling coordination level between agricultural trade and production in all provinces was generally low in 2010: only Shandong and Guangdong achieved primary coordination, while others remained in marginal coordination, marginal disorder, or low disorder states (running-in or antagonistic stages). By 2023, the coupling coordination degree had steadily improved across provinces. Only nine provinces (Ningxia, Shanxi, Gansu, Hainan, Jilin, Shaanxi, Jiangxi, Xinjiang, and Chongqing) lingered in low disorder; most provinces advanced to marginal disorder or marginal coordination. Notably, Fujian, Jiangsu, and Zhejiang reached low coordination, while Shandong and Guangdong attained moderate coordination. Furthermore, Shandong, Guangdong, and Fujian consistently ranked among the top three in coupling coordination level throughout most years. In contrast, central–western provinces such as Ningxia, Shanxi, Gansu, Hainan, Tibet, Shaanxi, Xinjiang, and Chongqing remained in the antagonistic stage of low disorder. This may be attributed to their relatively weak economic foundations, relative scarcity of agricultural resources, and the ineffective implementation of certain policies, all of which constrained the coupled development of agricultural trade and production.

### 4.3. Spatial Autocorrelation Analysis of Coupling Coordination Between Chinese Agricultural Trade and Production

To examine the spatial autocorrelation of the coupling and coordinated development of Chinese agricultural trade and production during the study period, this paper further calculates the Global Moran’s I index of the coupling coordination degree between agricultural trade and production. The specific results are shown in Table 5. It is evident that from 2010 to 2023, the Global Moran’s I indices for the coupling coordination degree of agricultural trade and production in China’s 31 provinces were all positive at the 1% significance level, indicating a significant positive spatial autocorrelation. This suggests that the coordinated development of agricultural trade and production in adjacent provinces mutually influences each other. In terms of specific values, the Global Moran’s index shows a fluctuating downward trend from 2010 to 2023, decreasing from 0.293 to 0.280. This suggests that the agglomeration level of agricultural trade and production in China gradually weakens over time. Additionally, the Moran’s I values across the years remained relatively stable, mostly ranging between 0.28 and 0.34, indicating a consistent spatial autocorrelation in the coupling coordination degree of China’s agricultural trade and production.

**Figure 5:**
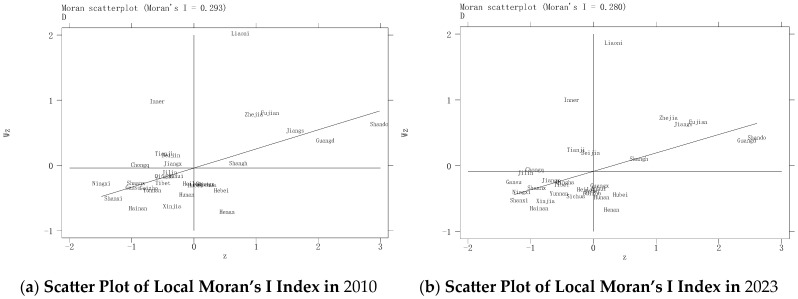
Local Moran’s I scatterplots of coupling coordination degree between agricultural trade and production in China. (**a**) Scatter Plot of Local Moran’s I Index in 2010. (**b**) Scatter Plot of Local Moran’s I Index in 2023.

To analyze the localized spatial clustering characteristics of the coupling coordination degree between China’s agricultural trade and production, this study computed the Local Moran’s I for the coupling coordination degree of 31 provinces in 2010 and 2023, visualized through Moran scatterplots (Figure 5). The Local Moran’s index distribution typically divides agglomeration situations into four quadrants: the first quadrant (high–high clustering), where both the province itself and its neighboring provinces have high coupling coordination degrees of agricultural trade and production; the third quadrant (low-low clustering), where both the province itself and its neighboring provinces have low coupling coordination degrees; the second quadrant (low–high clustering), where the province itself has a low coupling coordination degree but is surrounded by high-value provinces; and the fourth quadrant (high–low clustering), where the province itself has a high coordination degree but is surrounded by provinces with lower levels. The results show that the Local Moran’s indices of most provinces are located in the first (high–high clustering) and third quadrants (low–low clustering), indicating that the coupling coordination degree of agricultural trade and production in most provinces exhibits positive spatial autocorrelation, further verifying the global positive autocorrelation. Specifically, Shandong, Guangdong, Jiangsu, Zhejiang, Fujian, Liaoning, and Shanghai persistently formed high–high clustering, suggesting that these seven provinces have relatively high degrees of coupling coordination in agricultural trade and production and play a driving role in promoting the coordinated development of neighboring provinces, forming a high-level development zone predominantly in Eastern China. In contrast, provinces in Western China, such as Ningxia, Shanxi, Gansu, Shaanxi, Xinjiang, and Tibet, consistently demonstrate low–low clustering. The low coordination development level among these provinces and their neighboring provinces leads to a low coupling coordination degree. Additionally, fewer and more scattered provinces fell into the second (low–high clustering) and fourth quadrants (high–low clustering), including Inner Mongolia, Henan, Hebei, Beijing, and Tianjin, highlighting significant disparities in coupling coordination between these provinces and their adjacent areas. Overall, the coupling coordination of China’s agricultural trade and production exhibits spatial heterogeneity, underscoring the need to strengthen inter-provincial collaboration. Future policymaking should prioritize promoting high-level regional coordination across different areas.

## 5. Conclusions and Countermeasures

### 5.1. Conclusions

Based on panel data from 31 Chinese provinces from 2010 to 2023, this study constructed an evaluation index system to measure agricultural trade and production development levels, applied the coupling coordination degree model to assess their coupling coordination, and utilized spatial autocorrelation models to analyze the spatial autocorrelation characteristics of their coordinated development. The conclusions are as follows: (1) In terms of development levels, the development levels of China’s agricultural trade and production displayed an upward trend from 2010 to 2023. Agricultural trade developed significantly more slowly than agricultural production but had better momentum. Regional disparities were also observed, with both indicators showing higher levels in Eastern China and lower levels in western regions. (2) Regarding coupling coordination, the degree between China’s agricultural trade and production showed a steady upward trend from 2010 to 2023, indicating their interaction strengthened year by year. However, the overall coupling coordination level remained low, with an average value of only 0.445, consistently remaining in the marginal disorder running-in stage without transitioning into a coordinated stage, signifying vast room for improvement. Spatially, the coupling coordination degree also displayed regional heterogeneity, featuring a distinct stepwise pattern: Eastern China > Northeast China > Central China > Western China. Overall, the spatial pattern of “high in the east, low in the west” persists. Notably, Shandong and Guangdong reached moderate coordination, while Fujian, Jiangsu, and Zhejiang attained low coordination, and most other provinces lingered in marginal disorder or marginal coordination. (3) In terms of spatial pattern, the Global Moran’s I for the coupling coordination degree was positive and exhibited a fluctuating downward trend from 2010 to 2023, decreasing from 0.293 to 0.280, indicating a significant positive spatial autocorrelation clustering feature, but this clustering effect weakened over time. Looking at the Local Moran’s index, most regions were located in the first (high–high clustering) and third quadrants (low–low clustering), with fewer and more scattered regions in the second (low–high clustering) and fourth quadrants (high–low clustering). This suggests stable spatial clustering characteristics of high–high and low–low agglomeration in the coupling coordination degree between China’s agricultural trade and production. (4) Future research directions could be deepened from the dual perspective of international supply chain resilience and the Sustainable Development Goals (SDGs). On the one hand, from the perspective of international supply chain resilience, it is necessary to further explore the impact pathways of international grain trade fluctuations (such as the Russia–Ukraine conflict, the Red Sea crisis, etc.) on China’s agricultural trade and production coupling system. On the other hand, in terms of SDGs integration, the association modeling between the coupling coordination degree and indicators such as zero hunger (SDG2) and responsible consumption and production (SDG12) could be explored.

### 5.2. Countermeasures

The first countermeasure is to improve the coordination mechanism of agricultural trade and production from the perspective of food security and SDGs. We must further raise awareness and place high importance on the issue of low coupling coordination between the development of agricultural trade and production. At the national level, we need to focus on the fact that agricultural trade development lags behind agricultural production development. While adhering to the principle of moderate imports, we should strengthen the trade system from three dimensions: scale, structure, and security. Specifically, we should deeply integrate the global food governance system, promote a diversified strategy for agricultural imports, and continue to enhance and improve the emergency rice reserve mechanism among ASEAN countries and China–Japan–Korea to better align with the SDG2 goal of zero hunger.

It is crucial to coordinate domestic production and trade by establishing a dynamic monitoring system for domestic and international agricultural production, circulation, and consumption. We should comprehensively consider the country’s agricultural production capacity, food security strategy goals, and the requirements of the SDGs. Following the principle of “actively importing what is necessary, firmly blocking what is not, and striving to export what we excel in”, we should proactively import land-intensive agricultural products in which we do not have a comparative advantage, as well as high-quality products such as meat, eggs, and dairy. We should also reduce the import scale of agricultural products with high carbon footprints to meet the requirements of SDG13 on climate action and prioritize increasing the import share of organic agricultural products to align with the concept of responsible consumption and production under SDG12. Meanwhile, we should actively promote the export of advantageous agricultural products such as vegetables and fruits, strengthen branding and market-oriented development, and extend the industry chain to stabilize export growth. Strict management of import quotas for important agricultural products like wheat and rice is necessary, enhancing the “firewall” function of tariff quotas, controlling the import pace, and improving the early warning system for agricultural industry damage and the trade remedy system. This will help avoid potential damage to the agricultural industry from trade and thus coordinate the relationship between agricultural trade and production, promoting their coordinated and mutually reinforcing development and laying a solid foundation for achieving food security and the SDGs.

The second countermeasure is to implement differentiated regional coordinated development strategies. Research findings indicate significant regional disparities in agricultural trade, production, and their coupled coordination relationship. Against the backdrop of China’s vigorous promotion of its regional coordinated development strategy, it is necessary to fully consider the resource endowments, economic foundations, and levels of agricultural economic development of each region and implement differentiated regional coordination strategies in a timely manner to achieve the relatively balanced coordinated development of agricultural trade and production in various regions. Specifically, Eastern China, which is relatively advanced in development, should rely on free trade zones and cross-border e-commerce comprehensive pilot zones to focus on building international agricultural trade hubs, explore the “digital trade + high value-added processing” model, and connect with regional agreements such as the Regional Comprehensive Economic Partnership (RCEP) to enhance the ability to allocate global resources. For Central and Western China, efforts should be made to increase fiscal transfer payments and tax incentives, focusing on the construction of trade infrastructure such as cold chain logistics and storage facilities to narrow the infrastructure gap with the eastern region. In addition, cross-regional linkages and global cooperation should be further strengthened. Provinces and cities should enhance inter-regional economic exchanges, fully leverage the positive spillover effects of coupling coordination in Eastern China, and promote the establishment of “trade–production” alliances between Eastern China and Central, Western, and Northeastern China. The “east–west industrial enclave” system should be explored to promote the regional coordinated development of agricultural trade and production through inter-provincial cooperation and complementary advantages. At the same time, based on the background of global value chain restructuring and relying on free trade agreements such as the RCEP, measures such as promoting the mutual recognition of carbon labels and optimizing “green box” policies for food security should be taken to deepen agricultural trade cooperation with regions such as ASEAN and the EU. Through the above measures, not only can domestic regional coordinated development be promoted but participation in global food security governance can also be enhanced, ultimately achieving the coordinated progress of agricultural trade and production at both regional and global levels.

## Figures and Tables

**Table 1:** Agricultural trade and production index system.

System	Primary Indicator	Secondary Indicator	Measurement Method	Attribute
Agricultural Trade	Trade Scale	Total Trade Value	Directly Acquired	+
		Export Value	Directly Acquired	+
		Import Value	Directly Acquired	+
		Trade Balance	Export Value − Import Value	+
	Trade Structure	Processed Agricultural Product Export Share	Processed Agricultural Export Value/Total Export Value	+
		Agricultural Sideline Product Export Share	Agricultural Sideline Export Value/Total Export Value	+
		Primary Product Import Share	Primary Product Import Value/Total Import Value	+
		Animal Product Import Share	Animal Product Import Value/Total Import Value	+
		Export Market Diversification	$D_{it}=1-\sum_{j=1}^{N}{\left(\frac{export_{ijn}}{export_{in}}\right)}^{2}$, where $\frac{export_{ijn}}{export_{in}}$ Share of exports of product n from region i to country j in region i total exports	+
		Import Market Diversification	$D_{it}=1-\sum_{j=1}^{N}{\left(\frac{import_{ijn}}{import_{in}}\right)}^{2}$, where $\frac{import_{ijn}}{import_{in}}$ Share of import n from region i to country j in region i total imports	+
	Trade Competitiveness	Import Market Diversification	(Regional Export Value − Regional Import Value)/(Regional Export Value + Regional Import Value)	+
		Export Market Share	Regional Export Value/National Export Value	+
		Import Market Share	Regional Import Value/National Import Value	+
Agricultural Production	Production Scale	Agricultural Land Area	Directly Acquired	+
		Agricultural Water Usage	Directly Acquired	
		Agricultural Labor Force	Directly Acquired	+
		Total Agricultural Machinery Power	Directly Acquired	+
		Agricultural R&D Investment	(Gross Agricultural Output/GDP) × Government Science and Technology Expenditure	+
		Gross Output Value of Agriculture, Forestry, Animal Husbandry, and Fisheries	Directly Acquired	+
		Value-added of Agriculture, Forestry, Animal Husbandry, and Fishery	Directly Acquired	+
		Grain Output	Directly Acquired	+
		Fruit and Vegetable Output	Directly Acquired	+
		Meat, Egg, and Dairy Output	Directly Acquired	+
		Aquatic Product Output	Directly Acquired	+
	Production Structure	Cash Crop Sown Area Ratio	Cash Crop Sown Area/Total Crop Sown Area	+
		Forestry Output Share	Forestry Output Value/Gross Output Value of Agriculture, Forestry, Animal Husbandry, and Fisheries	+
		Animal Husbandry Output Share	Animal Husbandry Output Value/Gross Output Value of Agriculture, Forestry, Animal Husbandry, and Fisheries	+
		Fishery Output Share	Fishery Output Value/Gross Output Value of Agriculture, Forestry, Animal Husbandry, and Fisheries	+
	Production Efficiency	Land Productivity	Gross Agricultural Output Value/Crop Sown Area	+
		Labor Productivity	Gross Output Value of Agriculture, Forestry, Animal Husbandry, and Fisheries/Primary Industry Employment	+
		Resource Utilization Efficiency	Gross Output Value of Agriculture, Forestry, Animal Husbandry, and Fisheries/Fertilizer Usage	+

**Table 2:** Sensitivity analysis of weights in the coupling coordination degree model.

*α* = 0.5, *β* = 0.5		*α* = 0.4, *β* = 0.6		*α* = 0.3, *β* = 0.7		*α* = 0.6, *β* = 0.4		*α* = 0.7, *β* = 0.3	
Year	Nationwide	Nationwide	Volatility%	Nationwide	Volatility%	Nationwide	Volatility%	Nationwide	Volatility%
2010	0.413	0.420	1.88	0.428	3.68	0.404	2.02	0.396	4.25
2011	0.420	0.428	1.83	0.435	3.57	0.412	1.98	0.403	4.16
2012	0.427	0.435	1.88	0.442	3.66	0.418	2.02	0.409	4.25
2013	0.430	0.439	1.93	0.447	3.76	0.422	2.07	0.413	4.35
2014	0.434	0.442	1.99	0.450	3.89	0.424	2.14	0.415	4.50
2015	0.438	0.446	1.82	0.453	3.55	0.429	1.97	0.420	4.15
2016	0.444	0.451	1.67	0.458	3.25	0.436	1.80	0.428	3.79
2017	0.443	0.451	1.71	0.458	3.33	0.435	1.85	0.426	3.89
2018	0.447	0.455	1.79	0.462	3.48	0.438	1.93	0.429	4.06
2019	0.452	0.461	1.94	0.469	3.79	0.443	2.09	0.433	4.40
2020	0.458	0.467	1.89	0.475	3.68	0.449	2.03	0.440	4.28
2021	0.467	0.476	1.79	0.484	3.48	0.458	1.93	0.449	4.05
2022	0.479	0.487	1.57	0.494	3.05	0.471	1.70	0.463	3.57
2023	0.477	0.486	1.80	0.494	3.50	0.468	1.94	0.459	4.09

**Table 3:** Degree of coupling coordination and evaluation criteria.

Range of Coupling Coordination Degree	Qualitative Descriptor	Coupling and Coordination Stage
[0.0–0.1]	Extreme disorder	Antagonistic Stage
[0.1–0.2]	Serious disorder	
[0.2–0.3]	Moderate disorder	
[0.3–0.4]	Low disorder	
[0.4–0.5]	Marginal disorder	Running-in Stage
[0.5–0.6]	Marginal coordination	
[0.6–0.7]	Low coordination	
[0.7–0.8]	Moderate coordination	Coordinated Stage
[0.8–0.9]	Good coordination	
[0.9–1.0]	High coordination	

**Table 4:** Coupling coordination degree of agricultural trade and production in China’s provinces from 2010 to 2023.

Province	2010	2011	2012	2013	2014	2015	2016	2017	2018	2019	2020	2021	2022	2023	Mean
Anhui	0.386	0.394	0.390	0.391	0.398	0.408	0.431	0.453	0.472	0.492	0.501	0.502	0.496	0.487	0.443
Beijing	0.378	0.385	0.428	0.433	0.449	0.436	0.443	0.445	0.492	0.548	0.510	0.515	0.496	0.472	0.459
Fujian	0.525	0.543	0.553	0.566	0.571	0.596	0.611	0.609	0.621	0.624	0.638	0.665	0.679	0.672	0.605
Gansu	0.322	0.320	0.318	0.323	0.324	0.321	0.339	0.320	0.318	0.321	0.333	0.320	0.327	0.330	0.324
Guangdong	0.607	0.616	0.624	0.626	0.641	0.673	0.679	0.689	0.697	0.709	0.720	0.728	0.754	0.762	0.681
Guangxi	0.429	0.443	0.464	0.461	0.468	0.452	0.481	0.467	0.458	0.445	0.453	0.469	0.475	0.489	0.461
Guizhou	0.345	0.378	0.413	0.403	0.406	0.421	0.423	0.439	0.468	0.449	0.438	0.428	0.474	0.475	0.426
Hainan	0.329	0.332	0.333	0.329	0.332	0.337	0.341	0.337	0.333	0.325	0.316	0.333	0.366	0.376	0.337
Hebei	0.454	0.454	0.455	0.466	0.464	0.462	0.468	0.458	0.450	0.446	0.461	0.469	0.480	0.475	0.462
Henan	0.462	0.470	0.476	0.475	0.470	0.469	0.494	0.485	0.486	0.486	0.484	0.499	0.499	0.511	0.483
Heilongjiang	0.410	0.411	0.423	0.436	0.441	0.428	0.444	0.407	0.431	0.444	0.448	0.445	0.457	0.464	0.435
Hubei	0.415	0.429	0.438	0.452	0.452	0.454	0.480	0.460	0.447	0.453	0.475	0.508	0.531	0.528	0.466
Hunan	0.402	0.406	0.388	0.404	0.401	0.417	0.395	0.442	0.434	0.448	0.460	0.466	0.479	0.493	0.431
Jilin	0.376	0.385	0.388	0.393	0.372	0.359	0.376	0.345	0.347	0.345	0.360	0.342	0.359	0.351	0.364
Jiangsu	0.563	0.560	0.558	0.553	0.565	0.580	0.620	0.596	0.606	0.603	0.611	0.623	0.644	0.644	0.595
Jiangxi	0.381	0.368	0.396	0.371	0.370	0.373	0.376	0.367	0.367	0.365	0.370	0.395	0.404	0.398	0.379
Liaoning	0.482	0.491	0.500	0.511	0.508	0.519	0.515	0.520	0.510	0.513	0.504	0.492	0.508	0.515	0.506
Inner Mongolia	0.360	0.355	0.376	0.396	0.393	0.390	0.369	0.389	0.393	0.404	0.423	0.430	0.443	0.439	0.397
Ningxia	0.275	0.272	0.286	0.280	0.283	0.279	0.284	0.291	0.311	0.285	0.302	0.319	0.333	0.344	0.296
Qinghai	0.368	0.432	0.384	0.369	0.392	0.415	0.328	0.417	0.431	0.434	0.433	0.455	0.458	0.424	0.410
Shandong	0.688	0.711	0.720	0.732	0.738	0.731	0.738	0.734	0.733	0.743	0.753	0.769	0.778	0.781	0.739
Shanxi	0.293	0.291	0.302	0.344	0.353	0.342	0.347	0.329	0.292	0.295	0.323	0.336	0.360	0.339	0.325
Shaanxi	0.326	0.359	0.347	0.357	0.357	0.376	0.377	0.377	0.368	0.341	0.365	0.372	0.384	0.372	0.363
Shanghai	0.478	0.477	0.473	0.474	0.485	0.499	0.502	0.530	0.534	0.545	0.554	0.552	0.555	0.562	0.516
Sichuan	0.432	0.427	0.435	0.440	0.436	0.446	0.441	0.428	0.440	0.442	0.457	0.453	0.452	0.444	0.441
Tianjin	0.368	0.381	0.388	0.399	0.404	0.405	0.391	0.409	0.412	0.440	0.471	0.460	0.458	0.445	0.417
Tibet	0.367	0.362	0.376	0.362	0.358	0.355	0.365	0.349	0.338	0.372	0.329	0.403	0.449	0.418	0.372
Xinjiang	0.380	0.367	0.383	0.376	0.376	0.367	0.382	0.366	0.378	0.380	0.378	0.383	0.376	0.388	0.377
Yunnan	0.351	0.352	0.355	0.363	0.370	0.398	0.419	0.417	0.410	0.421	0.441	0.430	0.410	0.413	0.397
Zhejiang	0.501	0.514	0.512	0.513	0.515	0.529	0.542	0.546	0.555	0.559	0.569	0.584	0.604	0.617	0.547
Chongqing	0.333	0.343	0.345	0.346	0.345	0.335	0.362	0.309	0.311	0.340	0.333	0.340	0.367	0.368	0.341

**Table 5:** Global Moran’s I of the coupling coordination degree between China’s agricultural trade and production.

Year	Global Moran’s I	Z-Score	*p*-Value
2010	0.293	3.693	0.000
2011	0.294	3.731	0.000
2012	0.316	3.998	0.000
2013	0.333	4.196	0.000
2014	0.323	4.072	0.000
2015	0.324	4.051	0.000
2016	0.280	3.521	0.000
2017	0.302	3.751	0.000
2018	0.298	3.696	0.000
2019	0.314	3.871	0.000
2020	0.302	3.734	0.000
2021	0.273	3.425	0.001
2022	0.281	3.531	0.000
2023	0.280	3.509	0.000

Note: Global Moran’s I > 0 indicates positive spatial autocorrelation (agglomeration of similar values), Global Moran’s I < 0 indicates negative spatial autocorrelation (agglomeration of dissimilar values), and Global Moran’s I = 0 indicates a random distribution.

## Data Availability

The original contributions presented in the study are included in the article, further inquiries can be directed to the corresponding author.
